# Efficacy of Quercetin and Quercetin Loaded Chitosan Nanoparticles Against Cisplatin-Induced Renal and Testicular Toxicity via Attenuation of Oxidative Stress, Inflammation, and Apoptosis

**DOI:** 10.3390/ph17101384

**Published:** 2024-10-17

**Authors:** Alaa F. Bakr, Riham A. El-Shiekh, Mohamed Y. Mahmoud, Heba M. A. Khalil, Mohammad H. Alyami, Hamad S. Alyami, Omneya Galal, Dina F. Mansour

**Affiliations:** 1 Department of Pathology, Faculty of Veterinary Medicine, Cairo University, Giza 12211, Egypt; 2 Department of Pharmacognosy, Faculty of Pharmacy, Cairo University, Cairo 11562, Egypt; riham.adel@pharma.cu.edu.eg; 3 Department of Toxicology and Forensic Medicine, Faculty of Veterinary Medicine, Cairo University, Giza 12211, Egypt; mohamed_yehia@cu.edu.eg; 4 Department of Veterinary Hygiene and Management, Faculty of Veterinary Medicine, Cairo University, Giza 12211, Egypt; heba.ali315@gmail.com; 5 Faculty of Veterinary Medicine, King Salman International University, South Sinai, Ras Sudr 43312, Egypt; 6 Department of Pharmaceutics, College of Pharmacy, Najran University, Najran 66462, Saudi Arabia; mhalmansour@nu.edu.sa; 7 Department of Pharmacology and Toxicology, Faculty of Pharmacy, Ahram Canadian University, Giza 12581, Egypt; ominagalal@gmail.com; 8 Department of Pharmacology, Medical Research and Clinical Studies Institute, National Research Centre, Cairo 12622, Egypt; dinafmansour@gmail.com; 9 Department of Pharmacy, Faculty of Pharmacy, Galala University, Attaka, Suez 43511, Egypt

**Keywords:** quercetin, QUE.NPs, cisplatin, IL-10, Bax, Bcl-2

## Abstract

Background/Objectives: Flavonoids, including quercetin, have attracted much attention due to their potential health-promoting effects. Methods: The current experiment aims to see whether quercetin (QUE) in nanoparticle form could mitigate testicular and renal toxicity caused by cisplatin (CIS) more effectively than normally formulated QUE. Rats were randomly treated with CIS alone or in combination with QUE or QUE.NPs (Quercetin-loaded chitosan nanoparticles) for 4 weeks. QUE and QUE.NPs were given orally (10 mg/kg, three times a week), while CIS was given intraperitoneally (2 mg/kg, twice a week). Results: Compared to QUE- and CIS + QUE.NP-treated rats, CIS exposure induced anxiety and emotional stress as well as promoted oxidative stress in both testicular and renal tissues. Moreover, CIS reduced serum testosterone levels and diminished testicular IL-10, as well as CIS-induced renal failure, as indicated by hypokalemia, and increased levels of creatinine, urea, sodium, IL-18, and KIM-1. Further, severe histological changes were observed in the testis and kidney of CIS-intoxicated rats. Regarding immunohistochemical staining, CIS significantly upregulated Bax, downregulated Bcl-2, and moderately enhanced PCNA expression. Conclusions: Our findings suggest that both QUE and QUE.NPs modulated emotional disturbance and improved testicular and renal functions via modulation of oxidation, inflammation, and apoptosis. However, QUE.NPs performed better than QUE-treated rats.

## 1. Introduction

Worldwide, cancer is the leading cause of death. In 2020, approximately 19.3 million cancer cases and nearly 10 million cancer mortalities were reported. Universal cancer cases are expected to be 28.4 million in 2040 (47% higher than 2020) [1]. Multiple chemotherapies destroy cancer cells and limit their growth [2]. Potent side effects of chemotherapy, including fatigue, gastrointestinal disturbance, neurotoxicity, nephrotoxicity, and infertility, are considered the chief drawbacks in cancer treatment [3,4,5].

Cisplatin (*cis*-diamminedichloroplatinum II) is the most common chemotherapeutic agent used for the treatment of various types of cancer, including squamous cell carcinoma [6], osteosarcoma [7], lung [8], breast [9], esophageal [10], renal [11], and testicular cancer [12]. Despite this, the clinical use of cisplatin induces several side effects during treatment, including renal and testicular dysfunction, through the regulation of several pathways, even with low-dose administration [13].

Up to now, despite multiple research, no intermediation effectively treats or hinders side effects involving kidney failure and testicular damage induced by cisplatin in cancer patients [14,15]. Hence, several active principles extracted from herbs, including quercetin, are evaluated for improving proper pharmacological therapy in combination with cisplatin [16].

Quercetin (QUE) is a flavonoid that exists in several foods, including tomatoes, onions, broccoli, capers, black tea, green tea, apples, berries, nuts, and medicinal herbs involving *Sambucus canadensis*, *Ginkgo biloba*, and *Hypericum perforatum* [17,18]. QUE exhibits multiple therapeutic properties that may enhance the immune system and diminish infection possibility [19]. These therapeutic properties include anti-inflammatory, antioxidant, antiviral, and anticarcinogenic activities [20,21,22]. The name for QUE, according to the nomenclature of the International Union of Pure and Applied Chemistry (IUPAC), is 3, 3′, 4′, 5, 7-pentahydroxyflvanone, which indicates the presence of the hydroxyl (OH) group linked to the 3, 3′, 4′, 5 and 7 positions.

QUE is absorbed from different parts of the gastrointestinal tract and metabolized in the small intestine, large intestine, liver, and kidney by enzymes and bacteria [23]. QUE is, in plasma, conjugated with glucuronide and/or sulfate [24]. Maximum concentrations of QUE and its metabolites were detected in the lung, liver, and kidney [25].

After oral administration of QUE, about 20% is absorbed, and approximately 90% is metabolized within one hour after intraperitoneal injection [26]. Additionally, QUE is characterized by high molecular weight and poor water solubility [27,28]. Accordingly, its hydrophobic nature and rapid metabolism diminish the bioavailability of QUE and hinder its therapeutic efficiency. Therefore, our study aimed to investigate the possibility of upgrading the efficiency of QUE by incorporating it into nanoform. Nanoparticle formulations, due to their small size and large surface area, can significantly enhance solubility and improve bioavailability. This allows for the formulation of low-dose, food-derived compounds such as quercetin, which may be effective in alleviating chronic diseases. Chitosan, in particular, is a commonly used polymer in drug delivery systems with a well-established safety profile [29]. Additionally, chitosan has been shown to possess antioxidant properties, attributed to its ability to donate hydrogen atoms [30].

## 2. Results

### 2.1. Characterization of Quercetin-Loaded Chitosan Nanoparticles (QUE.NPs)

**Figure 1:**
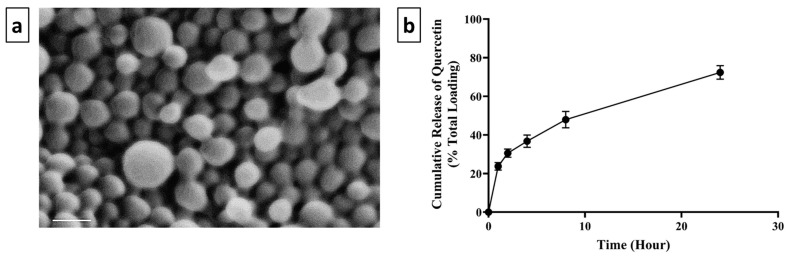
(**a**) SEM images of QUE.NPs. The scale bar represents 200 nm. Images represent at least three independent samples, with n > 500 NPs. (**b**) Cumulative release of quercetin from chitosan NPs over a 24 hr period. Data are presented as mean ± SD (n = 3).

The morphology of QUE.NPs is shown in Figure 1a. QUE.NPs demonstrated a spherical morphology, with an average unhydrated diameter of 112.13 ± 18.5 nm, compared to the average hydrated diameter of 294.7 ± 28.4 nm. Zeta potential and PDI of QUE.NPs were +34.5 ± 0.3 mV and 0.25 ± 0.02, respectively. Chitosan nanoparticles demonstrated a high quercetin loading of 86.4 ± 5.2%. Additionally, in vitro cumulative release of quercetin from chitosan NPs was in a burst pattern during the first few hours of incubation; 23.7 ± 1.9% of quercetin was released after 1 hr, then followed by a gradual release of 72.4 ± 3.5% of quercetin at 24 h of incubation (Figure 1b).

### 2.2. QUE and QUE.NPs Inhibit Anxiety and Emotional Disturbance Induced by Cisplatin

**Figure 2:**
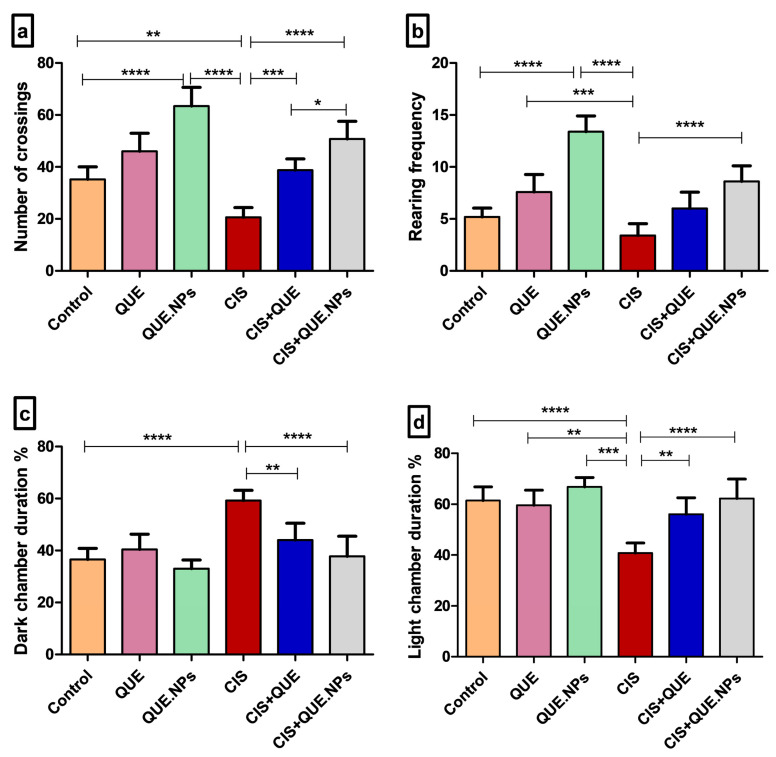
Effect of quercetin and its nanoform on the emotional behavior of cisplatin-intoxicated rats. (**a**) Open field test: number of crossing squares; (**b**) open field test: rearing frequency; (**c**) dark–light activity box: dark chamber duration %; and (**d**) dark–light activity box: light chamber duration %. Data are expressed as mean ± SEM (n = 7). Statistical difference: * *p* < 0.05, ** *p* < 0.01, and *** *p* < 0.001, **** *p* < 0.0001.

As depicted in Figure 2, in the open field test, CIS-treated rats showed a decrease in locomotor and exploratory activities expressed by a significant reduction in crossing squares and rearing episodes compared to controls. On the other hand, the administration of QUE and QUE.NPs reversed these behavioral alterations, as evidenced by a substantial increase in crossing squares and rearing episodes, which had a superior effect on the QUE.NP-treated rats compared to CIS-treated rats. Moreover, in the light–dark activity box, cisplatin-intoxicated rats displayed a marked increase in the duration percentage of the dark chamber, which was associated with a significant decrease in light chamber duration percentage compared to controls, while QUE and QUE.NP-treated rats exhibited a substantial increase in light chamber duration percentage, which was associated with a decrease in dark chamber duration percentage compared to CIS-treated rats.

### 2.3. QUE and QUE.NPs Counteract CIS-Induced Testicular and Renal Oxidative Stress

**Figure 3:**
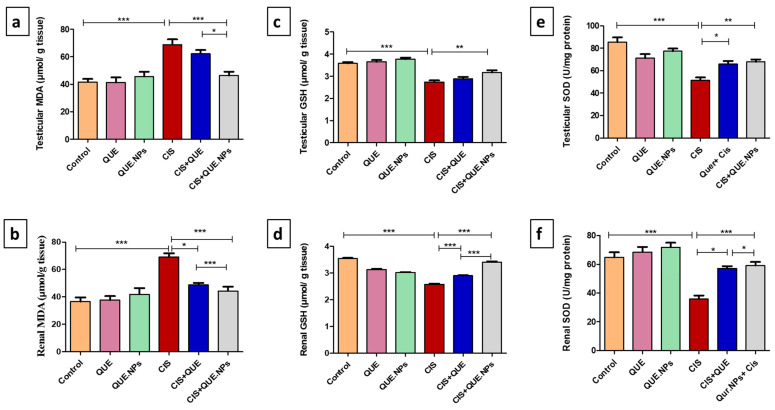
Effect of quercetin and its nanoform on oxidative stress induced by cisplatin in renal and testicular tissue of rats. (**a**) testicular MDA levels, (**b**) renal MDA levels, (**c**) testicular GSH levels, (**d**) renal GSH levels, (**e**) testicular SOD levels, and (**f**) renal SOD levels. Data are expressed as mean ± SEM (n = 7). Statistical difference: * *p* < 0.05, ** *p* < 0.01, and *** *p* < 0.001.

Cisplatin administration revealed substantial oxidative stress in testicular and renal tissues, confirmed by a significant elevation in MDA level (*p* < 0.0001) and a considerable reduction in both GSH and SOD activity as compared to the control group (*p* < 0.0001), indicating oxidative damage. Administration of QUE to CIS-treated rats resulted in a significant decrease in the renal MDA level (*p* < 0.05) and renal GSH (*p* < 0.001) compared to the CIS-treated group. However, the levels of these markers did not change in testicular tissue after QUE treatment. Regarding SOD levels, the CIS + QUE group exhibited a significant decrease in both testicular and renal tissue relative to the CIS group; concurrent treatment with CIS and QUE.NPs revealed a superior antioxidant effect in testicular and renal tissue compared to the QUE+CIS and CIS groups, as revealed in Figure 3.

### 2.4. QUE and Its Nanoform Mitigate the Reduction in Both Serum Testosterone and Testicular IL-10

**Figure 4:**
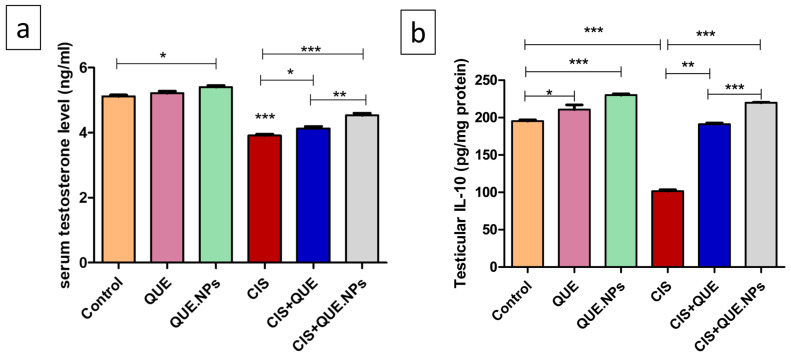
QUE and QUE.NPs mitigate cisplatin-induced reduction in serum testosterone and testicular IL-10. (**a**) serum testosterone level; (**b**) testicular IL-10 level. Data are expressed as mean ± SEM (n = 7). Statistical difference: * *p* < 0.05, ** *p* < 0.01, and *** *p* < 0.001.

Administration of cisplatin induced a significant reduction in testosterone levels (*p* < 0.001) compared to the normal control group (Figure 4). Concurrent treatment of QUE and QUE.NPs with cisplatin resulted in significant restoration of normal levels of testosterone compared to CIS and control groups (*p* < 0.05 and *p* < 0.01, respectively). It is worth mentioning that the administration of QNE.NPs alone in normal rats improved testosterone levels relative to the control group (*p* < 0.05). In addition, a significant reduction in testicular IL-10 level was recorded in the CIS group compared to the control group (*p* < 0.001). QUE and its nanoform significantly promoted IL-10 in testicular tissues compared to the CIS group (*p* < 0.01 and *p* < 0.001, respectively). Remarkably, a better anti-inflammatory effect was observed in CIS + QUE.NPs relative to the CIS + QUE group (*p* < 0.0001). Similarly, administration of either QUE or QUE.NPs alone improved the level of IL-10 significantly compared to the control group (*p* < 0.0001).

### 2.5. Sperm Concentration, Viability, and Morphology

**Figure 5:**
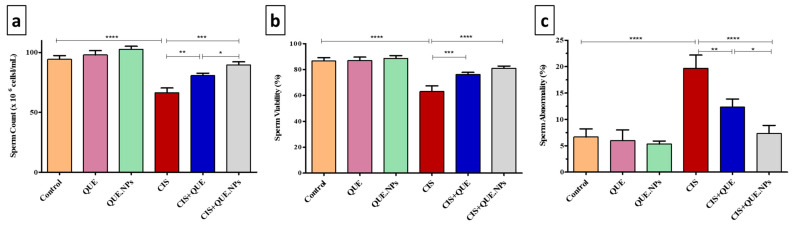
Evaluation of sperm count, viability, and morphology. (**a**) sperm count; (**b**) sperm viability; (**c**) sperm abnormality. Data are expressed as mean ± SEM (n = 7). Statistical difference: * *p* < 0.05, ** *p* < 0.01, *** *p* < 0.001, and **** *p* < 0.0001.

Cisplatin significantly (*p* < 0.0001) decreased the sperm count relative to control untreated rats; however, the administration of QUE and QUE.NPs alleviated the toxicity of cisplatin and showed a significant (*p* < 0.001 and *p* < 0.0001, respectively) increase in sperm count relative to cisplatin-treated rats. Additionally, QUE.NP-treated rats showed a substantial (*p* < 0.05) increase in sperm count relative to QUE-treated animals (Figure 5a).

Rats treated with CIS demonstrated a significant (*p* < 0.0001) reduction in sperm viability by ~24% relative to control rats, while QUE and QUE.NPs significantly (*p* < 0.001 and *p* < 0.0001, respectively) increased sperm viability when administrated with CIS compared to rats treated with CIS only (Figure 5b).

Evaluation of sperm morphology demonstrated that CIS-treated rats revealed a marked increase in sperm abnormality (*p* < 0.0001) compared to control rats; however, simultaneous administration of QUE or QUE.NPs with cisplatin significantly (*p* < 0.01 and *p* < 0.0001, respectively) decreased sperm abnormality relative to CIS-treated rats. Also, the administration of QUE.NPs with cisplatin significantly (*p* < 0.05) reduced sperm abnormality levels compared to the CIS + QUE group (Figure 5c).

### 2.6. QUE and QUE.NPs Attenuate CIS-Induced Kidney Damage

Rats treated with CIS exhibited a prominent alteration in kidney markers, as evidenced by hypokalemia (*p* < 0.001), in addition to high serum concentration of creatinine (*p* < 0.0001), urea (*p* < 0.001), and sodium (*p* < 0.001) compared to the control group. Concurrent administration of QUE with CIS significantly reduced serum creatinine and urea only (*p* < 0.05) and did not improve levels of electrolytes. Conversely, the CIS + QUE.NPs group exhibited a significant reduction in serum creatinine, urea, and sodium levels and alleviation of serum potassium levels compared to the cisplatin group and CIS + QUE group (Table 1).

### 2.7. QUE and QUE.NPs Adjust Interleukin-18 (IL-18) and Kidney Injury Molecule-1 (KIM-1) Levels in Renal Tissue

**Figure 6:**
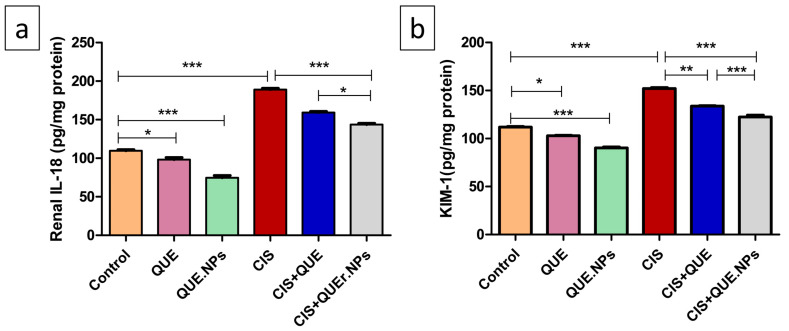
Quercetin and its nanoform amend renal interleukin-18 (IL-18) and kidney injury molecule-1 (KIM-1). (**a**) renal IL-18 level; (**b**) renal KIM-1 level. Data are expressed as mean ± SEM (n = 7). Statistical difference: * *p* < 0.05, ** *p* < 0.01, and *** *p* < 0.001.

A significant increase in renal IL-18 and KIM-1 was recorded in CIS-treated rats relative to the control group (*p* < 0.001). The combination of CIS with QUE significantly reduced the levels of IL-18 and KIM-1 compared to the CIS group (*p* < 0.05 and *p* < 0.01, respectively). Similarly, QUE.NPs extensively decreased levels of IL-18 and KIM-1 when administrated in combination with cisplatin (*p* < 0.001) and exhibited a better effect relative to the CIS + QUE group (*p* < 0.05 and *p* < 0.001, respectively). Unexpectedly, rats treated with either QUE or its loaded nanoform at the same dose revealed a significant decrease in IL-18 and KIM-1 levels compared to the control group (*p* < 0.05 and *p* < 0.001, respectively). These results pointed to the valuable reno-protective effect of QUE in both free and loaded nanoform (Figure 6).

### 2.8. QUE and QUE.NPs Protect the Histological Structure of the Testis and Kidney

**Figure 7:**
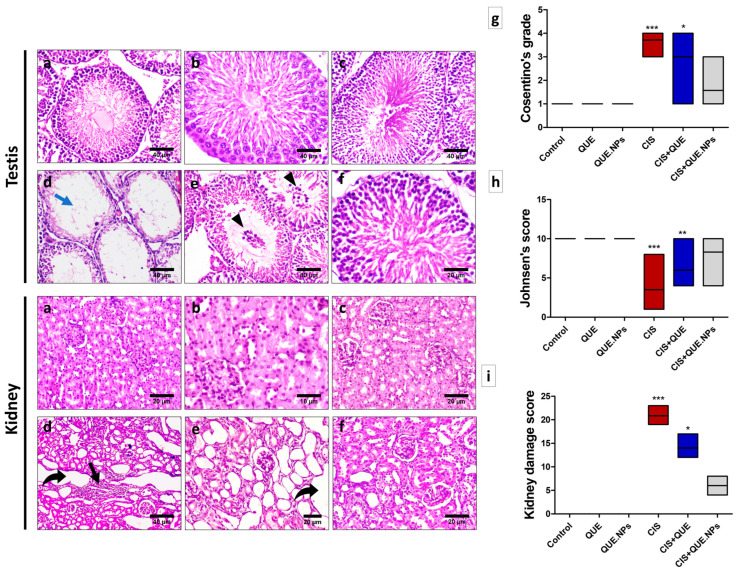
QUE and its nanoform attenuate CIS-induced apoptosis and promote cellular regeneration. (**a**) control group; (**b**) QUE group; (**c**) QUE.NPs group; (**d**) CIS group; (**e**) CIS + QUE group; (**f**) CIS + QUE.NPs group; (**g**) testicular damage score (Cosentino’s grade); (**h**) spermatogenesis score (Johnsen’s scoring); (**i**) kidney damage score. Data are expressed as median ± SEM. Statistical difference: * *p* < 0.05, ** *p* < 0.01, and *** *p* < 0.001. Remarkable marks shown in the figure are as follows: oligospermia (blue arrow), spermatid giant cells (arrowhead), interstitial nephritis (black arrow), and tubular dilatation with complete renal epithelial necrosis (curved arrow).

An examination of both testicular and renal tissues is depicted in Figure 7. The testicular tissues from the control, QUE, and QUE.NPs groups exhibited intact seminiferous tubules with normal spermatogenic cells and abundant sperms in the lumen. CIS-treated rats revealed significant atrophy and degeneration of seminiferous tubules with few spermatogenic cells and oligospermia, as well as congestion of blood vessels in interstitial space. In the CIS + QUE group, multiple spermatid giant cells, seminiferous tubular degeneration, and disorganized–atrophied germinal cells were detected. The histological structure of seminiferous tubules and the germ cells significantly improved in the CIS + QUE.NPs group. Numerical data revealed that there was a significant enhancement of histological damage of testicular tissues (Cosentino’s grade) and downregulation of spermatogenesis (Johnsen’s scoring) in the CIS group (*p* < 0.001) compared to the control group. Administration of CIS with QUE upgraded both histological damage (*p* < 0.05) and spermatogenesis (*p* < 0.01) compared to the control group. A marked upregulation in the histological score and spermatogenesis was recorded in the CIS + QUE.NPs group, and no statistical difference was recorded relative to the control group (Figure 7i,j).

Kidneys from control, QUE, and QUE.NPs rats revealed normal glomerular and tubular histological structures. Administration of CIS exhibited severe histological deterioration of kidney tissue (*p* < 0.001) compared to the control group. These alterations included interstitial nephritis, glomerular atrophy, tubular dilatation, cell degeneration, and necrosis. Concurrent administration of QUE and QUE.NPs with cisplatin upgraded the histological structure, especially in the CIS + QUE.NPs group. It is worth mentioning that neither QUE nor QUE.NPs alone induced any alteration in both renal and testicular tissues.

**Figure 8:**
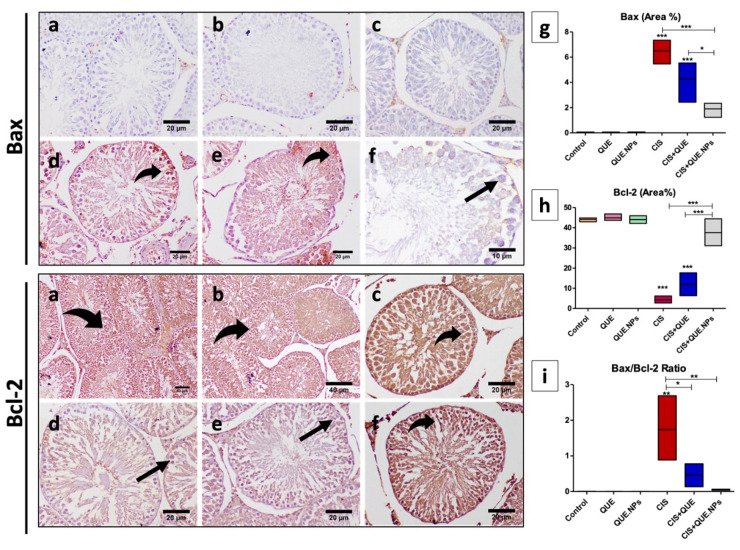
Immunohistochemical staining of testicular tissue with Bax and Bcl-2. (**a**) control group; (**b**) QUE group; (**c**) QUE.NPs group; (**d**) CIS group; (**e**) CIS + QUE group; (**f**) CIS + QUE.NPs group; (**g**) Bax (area%); (**h**) Bcl-2 (area%); (**i**) Bax/Bcl-2 ratio. Data are expressed as mean ± SEM (n = 7). Statistical difference: * *p* < 0.05, ** *p* < 0.01, and *** *p* < 0.001. Remarkable marks shown in the figure are as follows: strong expression (curved arrow) and weak expression (black arrow).

**Figure 9:**
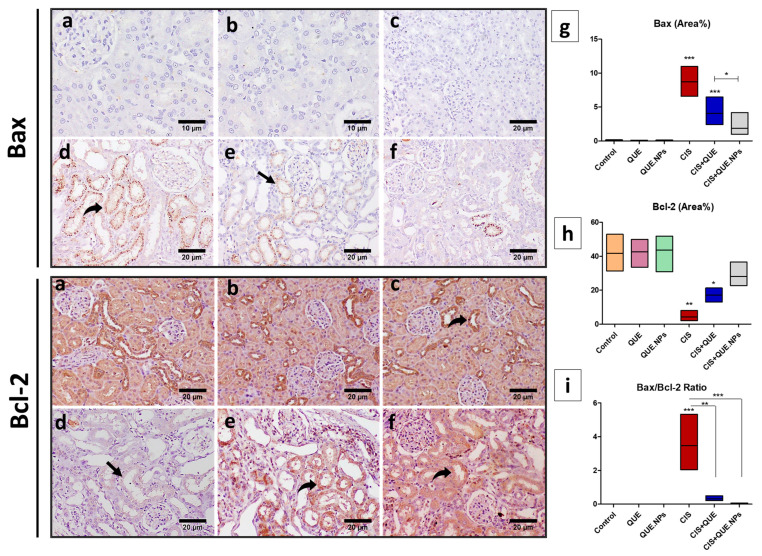
Immunohistochemical staining of renal tissue with Bax and Bcl-2. (**a**) control group; (**b**) QUE group; (**c**) QUE.NPs group; (**d**) CIS group; (**e**) CIS + QUE group; (**f**) CIS + QUE.NPs group; (**g**) Bax (area%); (**h**) Bcl-2 (area%); (**i**) Bax/Bcl-2 ratio. Data are expressed as mean ± SEM (n = 7). Statistical difference: * *p* < 0.05, ** *p* < 0.01, and *** *p* < 0.001. Remarkable marks shown in the figure are as follows: strong expression (curved arrow) and weak expression (black arrow).

The expression of Bax (pro-apoptotic marker) was significantly increased (*p* < 0.001) in testicular and renal tissues of CIS and CIS + QUE groups as determined by increasing the intensive brown staining, while QUE.NPs downregulate the expression of Bax that the administration of cisplatin induces. Conversely, the expression of Bcl-2 (anti-apoptotic marker) in renal and testicular tissues was diminished in the CIS group, while treatment with either free QUE or QUE.NPs ameliorated its expression (Figure 8 and Figure 9).

Considering the BAX/Bcl-2 ratio, a high Bax/Bcl-2 ratio points to the sensibility of cells to apoptosis, as recorded in renal and testis tissues of CIS-treated rats (*p* < 0.001 and *p* < 0.01, respectively). On the contrary, a low Bax/Bcl-2 ratio indicated resistant cells, as recorded in renal and testis tissues of CIS + QUE rats (*p* < 0.01 and *p* < 0.05, respectively) and CIS + QUE.NPs rats (*p* < 0.001 and *p* < 0.01, respectively) compared to the CIS group.

**Figure 10:**
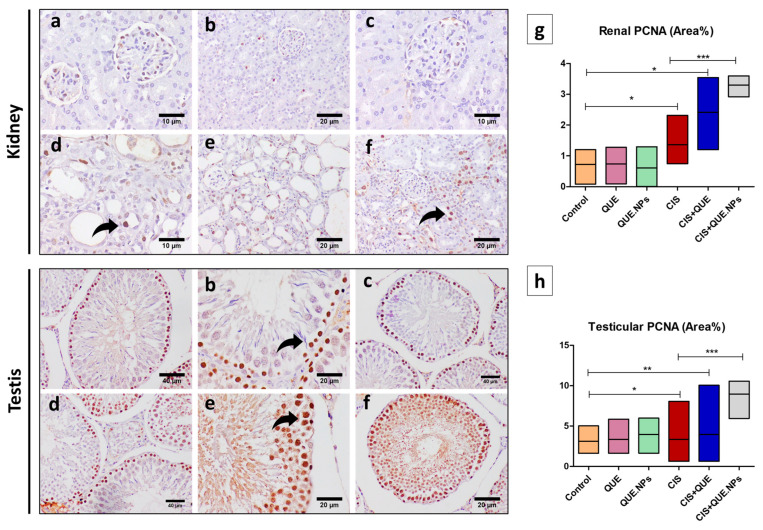
Immunohistochemical staining of renal and testicular tissue with PCNA. (**a**) control group; (**b**) QUE group; (**c**) QUE.NPs group; (**d**) CIS group; (**e**) CIS + QUE group; (**f**) CIS + QUE.NPs group; (**g**) renal PCNA (area%); (**h**) testicular PCNA (area%); Data are expressed as mean ± SEM (n = 7). Statistical difference: * *p* < 0.05, ** *p* < 0.01, and *** *p* < 0.001. Remarkable marks shown in the figure are as follows: strong expression (curved arrow) and weak expression (black arrow).

Subsequently, the proliferative capability of testicular and renal tissue was evaluated by PCNA immunohistochemical staining. The intensity of expression was significantly increased in the CIS group in both renal and testicular tissue compared to the control group (*p* < 0.05); concurrent treatment with QUE or QUE.NPs significantly augmented PCNA expression in testicular and renal cells of the rats treated with cisplatin (Figure 10). Therefore, the results showed that QUE in both free and loaded nanoform stimulated proliferation capacity under CIS exposure.

## 3. Discussion

Quercetin is a commonly occurring flavonoid in plants and is well known for its several beneficial activities: as an antioxidant, anti-inflammatory, cardiovascular protective, and anti-oncology agent [31]. This research is intended to offer some insights and inspirations for the scientific basis for its application in clinical practice to lessen the toxicity of the drugs. It is worth highlighting that flavanols are the most dominant flavonoids in fruits and vegetables; quercetin is the most ingested in the human diet.

Cisplatin is considered the most used antitumor agent in treating several cancers; however, its application is restricted because of its side effects on multiple organs [32]. Renal and testicular dysfunction are the most confirmed after-effects of CIS treatment [33,34,35]. In our study, we investigated the harmful effects of cisplatin on the testis and kidney and the possible modulating effect of QUE in both free and loaded nanoforms.

QUE.NPs showed a small particle size with high loading efficiency, which may be attributed to the mass ratio of chitosan to sodium tripolyphosphate of 3:1 during synthesis that may result in more vital intramolecular interaction of chitosan [36,37]. Also, in NP synthesis procedures, using chitosan solution with an adjusted pH of 5 may result in the homogeneity and the small size of nanoparticles, whereas a pH of more than 5.5 probably produces microparticles with heterogeneous distribution [38,39]. A positive zeta potential charge with respect to QUE.NPs confirms nanoparticle stability and minimizes aggregation probability [40]. The positive zeta potential of QUE.NPs results from amino groups of chitosan molecules on the nanoparticle surface [41,42].

Cisplatin-treated rats showed a decrease in general motor activity and increased anxiety, as visualized by a marked increase in the dark chamber duration percentage compared to controls. These results agreed with the previous study of Abdollahzadeh et al. [43]. On the other hand, the administration of CIS with QUE or QUE.NPs alleviated this emotional disturbance, as expressed by a marked increase in general motor activity and light chamber duration percentage [44]. The superior effect of QUE.NPs over QUE may be attributed to upgrade their bio-efficacy and the bioavailability. Open field and dark–light activity boxes are standard tests used in assessing unconditioned anxiety in rodents. The open field test depends on the natural fear of rats to open areas. At the same time, the dark–light activity box is dependent on the natural fear of rodents to brightly illuminated areas [1]. Renal and testicular injuries are often associated with locomotion and emotional disturbance [2,3]. Also, a recent study demonstrates the behavioral dysfunction associated with cisplatin administration [4]. Moreover, Orabi et al. discussed the harmful effects of doxorubicin, a standard anticancer agent, on the neurobehavioral function of rats [5].

QUE is a beneficial flavonoid, but its pharmacological use is restricted due to its low oral bioavailability and hydrophobic nature [45]. Thus, the incorporation of QUE into a chitosan nanoparticle form was able to reverse the harmful effects of cisplatin.

The occurrence of testicular and renal toxicity after treatment with cisplatin is principally attributed to tempering the antioxidant defense mechanism and generating endogenous reactive oxygen species (ROS) [46]. Oxidative stress happens once the cell is incapable of counterbalancing the generated ROS. Subsequently, ROS begins controlling numerous signaling pathways involving MAPK, Nrf2, DNA damage, cellular apoptosis, and necrosis [47]. Additionally, cisplatin enhances the accumulation of ROS by interrupting the expression of antioxidant enzymes [48]. Accumulation of ROS in the cells of rats treated with cisplatin arrested the mitochondrial antioxidant competence, resulting in an imbalance of the mitochondrial redox status with enhanced apoptosis of renal and testicular tissues, a substantial drop in sperm count [49], and germ cell death [50]. This hypothesis is confirmed in our study by increasing MDA levels and decreasing both GSH and SOD activity in the testicular and renal tissue of the CIS group.

Administration of QUE in either free or nanoform to rats intoxicated with CIS normalized the levels of MDA, GSH, and SOD significantly. QUE is known as a strong–free radical scavenger and a metal chelator. Moreover, it can modulate the levels of MDA, SOD, CAT, and GSH [51,52]. In addition, QUE was found to potentiate the gene expression of GR under stress conditions that reutilize GSSG (oxidized glutathione disulfide) to GSH [53]. GSH, a hydrogen donor, coordinates with superoxide dismutase (SOD) to transform O^2−^ into H_2_O_2,_ followed by decomposition into non-toxic H_2_O [54]. It is worth mentioning that the phenyl ring of QUE has hydroxyl groups (–OH) on its sides that can bind to critical amino acids of two enzymes at active sites [55]. Along these lines, QUE can inhibit the effect of acetylcholinesterase (AChE) and butyrylcholinesterase (BChE), which have oxidative features [56]. Furthermore, QUE has been reported to promote mitochondrial biogenesis, dynamics, and structure besides enhancing the availability of endogenous antioxidants by modulating Nrf2, NRFB, AMPK, and MAPK signaling pathways [57,58,59].

Testosterone level is a critical marker for evaluation of testicular toxicity. A significant decline in serum testosterone following administration of CIS was recorded in our study. This finding is attributed to the blocking effect of CIS with the luteinizing hormone (LH) receptor, in addition to the downregulation of cytochrome P450 activity and the expression of steroidogenesis genes, including CYP19A1 and CYP17A1 [60,61,62]. Moreover, testosterone is highly sensitive to testicular oxidative stress induced by CIS, which results in damage to the otocell membrane [60], testosterone-producing Leydig cells, and all spermatogenic cell types [63], as confirmed by our histopathological and morphometric observations (increase in Cosentino grade and decrease in Johnsen scoring).

The spermatogenesis process occurs through the combination of the spermatogenic cells, interstitial cells of the testicular tissue, and autocrine cytokines, including interleukin 10 (IL-10) that is produced by Sertoli cells after stimulation by gonadotropins [64,65]. IL-10 is an anti-inflammatory cytokine that works against the elicitation of pro-inflammatory cytokines and leukocytes [66]. Furthermore, IL-10 is critical in protecting tissue homeostasis through immune responses [67]. Triggering the inflammatory cascade following CIS induction was proven previously by a significant diminution in the anti-inflammatory cytokine IL-10 level with enhanced levels of testicular TNF-α and IL-1β [68,69]. Of note, induction of the inflammatory cascades along with amplified creation of inflammatory cytokinin were attributed to excessive ROS generated by CIS, which augments gonad toxicity and spermato-toxicity [70].

In our study, concurrent treatment of CIS with QUE in either free or nanoform induced elevation of the testicular function and histological structure, confirmed by elevation of testosterone, IL-10 levels, and Johnsen scores, in addition to a reduction in Cosentino grade. This finding could be attributed to the capability of QUE to prevent damage from lipids, proteins, and DNA, which can induce a drop in semen parameters [71]. More to the point, QUE was reported previously to cause a dose-dependent normalization of 3β- Hydroxysteroid dehydrogenase (3β-HSD) and 17β-Hydroxysteroid dehydrogenase (17β-HSD) levels, which regulate the production of testosterone and testicular function [72]. In addition, QUE has an anti-inflammatory effect by hindering the production of inflammatory cytokines, blocking the active sites of lipoxygenase enzymes [73], and enhancing anti-inflammatory cytokines, including IL-10 [74].

CIS-induced nephrotoxicity has been verified in our present study, which is presented by a reduction in potassium levels and an elevation of creatinine, urea, sodium, IL-18, and KIM-1 levels. These alterations in biochemical parameters relate to histopathological results. Multiple histological deteriorations were detected, including interstitial nephritis, glomerular atrophy, tubular cell degeneration, and necrosis. Approximately 30% of cisplatin-treated patients suffer from nephrotoxicity, which is considered the main drawback of cisplatin chemotherapy [75]. Cisplatin causes a quick fall in renal function as it is concentrated and reabsorbed by tubular renal cells (five times more than in the blood) [12]. CIS accumulation in the kidney is a high density of negatively charged mitochondria localized in the proximal tubules, attracting positively charged CIS hydrolyzed complexes [76]. In addition, renal uptake and excretion of cisplatin are regulated with various transporters such as OCT2 and MATE1, which are localized in proximal renal tubules, resulting in inflammation, oxidative stress, cell death, and a marked reduction in renal function [33].

High expression of renal interleukin-18 (IL-18), a proinflammatory cytokine, and kidney injury molecule-1 (KIM-1), a transmembrane protein, is considered an early diagnostic marker for toxic or hypoxemic glomerular/tubular injury produced by damaged proximal renal tubules [77,78]. In particular, KIM-1 is augmented earlier than standard biomarkers, e.g., plasma creatinine, urea, and proteinuria [79].

Data from this study point to the protective role of quercetin in either free form or nanoform against CIS-induced nephrotoxicity. Added to the antioxidant effect, quercetin was found to regulate ion transporters and channels [80] as well as initiate Na^+^-K^+^-2Cl^−^ cotransporter 1 (NKCC1), which causes an increase in cytosolic Cl^−^ concentration and, in sequence, hinders Na^+^, K^+^-ATPase activity [81]. These actions explain the ability of QUE.NPs to reduce Na+ reabsorption and regulate renal function, as evidenced by a significant reduction in creatinine and urea levels and avoiding the hypokalemic effect and KIM-1 expression induced by cisplatin. Similarly, quercetin in free or nanoform downregulated the expression of NF-κB and TNF-α, suppressing the production of IL-18 in macrophages [82].

Stimulation of the mitochondria-dependent intrinsic apoptosis pathway occurs after the administration of cisplatin by interrupting the balance between anti-apoptotic (Bcl2) and pro-apoptotic (Bax) proteins [61]. To detect the mechanistic pathways of QUE and QUE.NPs, immunohistochemistry staining of testicular and renal tissue we performed with respect to CIS-induced apoptosis and different apoptosis-related proteins. There is an explicit agreement that CIS downregulated the transcript of Bcl2 by p53 stimulation and upregulated both Bax expression and the Bax/Bcl2 ratio [83,84,85], which corroborates the present study. These results illustrated the degeneration and apoptosis of seminiferous and renal tubules in our histopathological observation.

PCNA is a cell proliferation marker that has a critical role in regulating the life and death of a cell [86]. PCNA is expressed generally in spermatogonia and early-phase primary spermatocytes in all stages of the seminiferous tubules and renal tubular cells during regeneration [87]. The expression of this marker is induced by p53 and interrelates with several p53-dependent proteins involving p21. Once p53 is absent, the accumulation of PCNA occurs, and DNA replication launches [88]. Repositioning of PCNA and DNA repair is dependent on the blocking of cyclin D1 expression, considered the primary monitor of the G1 phase of the cell cycle, and its downregulation ends by cell cycle arrest [89]. In addition to this point, p21 was found to hinder the entrance of the damaged DNA to the cell cycle by restraining p53-dependent plus p53-independent apoptosis [90]. Taken together, CIS causes simultaneous amplification of p53, p21, and PCNA expression with a diminishing of cyclin D1 levels, putting a stop to the cell cycle, as verified by previous studies [88,90]. QUE stimulated the level of cyclin D1 in normal cells, not in tumor cells, which increased the levels of cellular p21 and Cdk2. All this ended with the stabilization of the cyclin D1/Cdk2/p21/proliferating cell nuclear antigen (PCNA) pathway and facilitated cell cycle proliferation [91], as confirmed in our study.

At the same dose level, the superior protective effect against cisplatin toxicity is attributed to the nanoform of quercetin compared to the free form. Moreover, QUE did not improve MDA, GSH, and electrolyte levels compared to QUE.NPs at the same dose. Preliminary analyses of the pharmacokinetic properties of quercetin in humans revealed a very poor bioavailability (~2%) after oral or intravenous injection at a dose of 8 to 2000 mg/m^2^. Moreover, QUE exhibited low solubility, oral absorption (3–17% at a dose of 100 mg), extensive metabolism, and speedy elimination [18,92]. Thus, previous studies recommended the optimization of the structure of QUE to develop a novel compound with better bioavailability and solubility [93].

**Figure 11:**
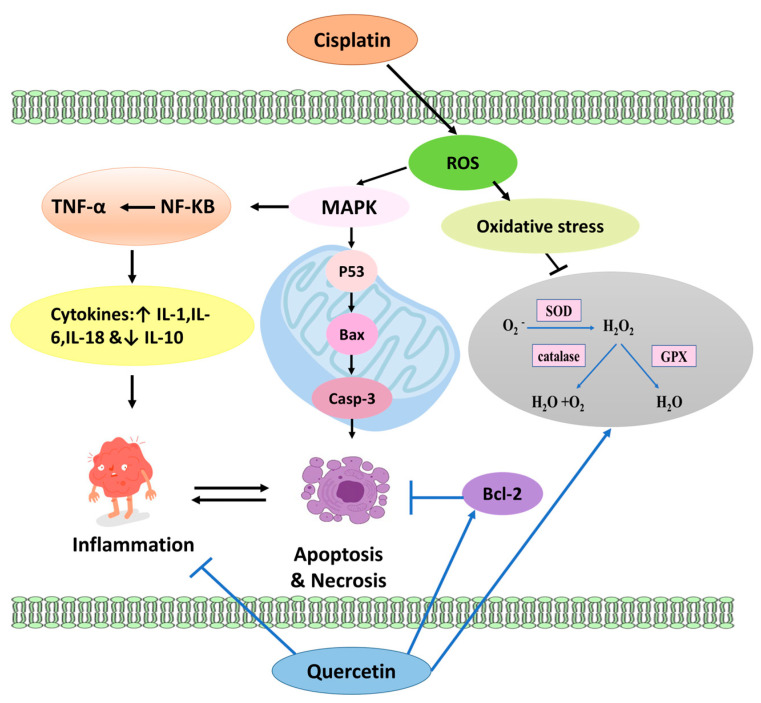
Modulation of oxidative stress and apoptotic pathways of CIS by quercetin. ROS, reactive oxygen species, and GSH, glutathione; MDA, malondialdehyde; SOD, superoxide dismutase; IL, interleukin; Casp–3, caspase 3; Bax, BCL–2-associated X protein; Bcl-2, B-cell lymphoma 2; TNF–α, tumor necrosis factor α; NF–κB, nuclear factor κB.

It should be pointed out that QUE supplementation diminishes inflammation, oxidative stress, and apoptosis in normal cells and does not hinder the anti-tumor action of CIS in cancer cells, as reported earlier [94]. The underlying pathways of CIS and QUE reviewed in the text are illustrated in Figure 11. Finally, it is worth mentioning that groups treated with either QUE or QUE.NPs alone did not induce any alteration in the biological parameters of treated rats. Further, QUE.NP-treated rats showed significant positive results compared to the control group and QUE in some parameters, including emotional behavior, renal testosterone, testicular IL-10, renal IL-18, and KIM-1. Various research has reported that dietary supplementation with 200–1200 mg of quercetin daily has a health-beneficial effect without any toxicity or change in biological parameters [95]. Accordingly, QUE.NPs could be prescribed at lower dosages than free-form QUE to improve their health status.

## 4. Materials and Methods

### 4.1. Plant Material

The aerial parts of *Hypericum perforatum* L. were obtained from Haraz herbal store, Cairo, Egypt, and checked by Dr. Mohamed El-Gibali, Senior Botanist at El-Orman Botanical Garden. A voucher specimen was deposited in the Herbarium of the Pharmacognosy Department, Faculty of Pharmacy, Cairo University (No. 2019-7-18).

### 4.2. Spectral Analysis

The ^1^H-NMR spectrum was recorded at 100 MHz in MeOH-*d_4_* using a Bruker high-performance digital NMR spectrometer (Karlsruhe, Germany) with tetramethylsilane (TMS) as the internal standard, and the chemical shifts were given in parts per million (ppm) relative to TMS.

TLC-MS was carried out using TLC silica gel 60 RP-18 F245s (Merck, Darmstadt, Germany) and methanol, with H_2_O (90:10) as a solvent system. Mass spectra for the required TLC spot were measured using Advion plate express with a compact mass spectrometer (Ithaca, NY, USA) in the positive and negative electrospray ionization (ESI) polarity modes.

### 4.3. Isolation of Quercetin

The air-dried powder (3 kg) was extracted with methanol, and the methanolic extract was evaporated till dry to yield a brown residue (550 g). The methanolic extract (300 g) was fractionated into a chloroform fraction (185 g) and *n*-butanol fraction (75 g). The *n*-butanol fraction was chromatographed on a polyamide column, starting with 100% distilled water (4 g), then 20% methanol (11 g), 40% methanol (22 g), 60% methanol (14 g), 80% methanol (10 g), and 100% methanol (13 g). The methanolic fraction (5 g) was further subjected to Sephadex and eluted with 50% methanol (15 mL was collected). Test tubes (24-33) were collected and yielded yellow powder. The powder was investigated on TLC gave one spot that was compared with authentic flavonoids by comparing R_f_ value The compound was matched with quercetin. The identity of the purified compound was elucidated by ^1^H NMR and thin-layer chromatography coupled with mass spectroscopy (TLC-MS).

ESI^+^ showed *m/z* 303.5 and 301.5 in ESI^-^, ^1^H-NMR (MeOH-*d4*, 400 MHz): *δ* 7.76 (1H, d, J = 2.1 Hz), 7.62 (1H, d, J = 2.1 Hz, J = 8.5 Hz), 6.91 (1H, d, J = 8.5 Hz), 6.39 (1H, d, J = 2.07 Hz), and 6.19 (1H, d, J = 2.07 Hz). The values were compared with previously published papers where the data agreed with [96].

### 4.4. Quercetin-Loaded Chitosan Nanoparticles Synthesis

Synthesis of QUE.NPs were accomplished using an ionic gelation technique [37,97,98]. In brief, 0.2% (*w*/*v*) chitosan (Mw~100:300 KDa, deacetylated degree 90%; ACROS ORGANICS^®^, Bristol, UK) was dissolved in dilute acetic acid (2% *v*/*v*) overnight at room temperature, and pH was adjusted to 5 using 1 M NaOH. The next day, quercetin solution (10 wt. % % of chitosan) and aqueous sodium tripolyphosphate (0.1%, *w*/*v*) were mixed before being added dropwise to the chitosan solution. The nanoparticle solution was stirred for 45 min. The NP solution was centrifuged for 20 min at 13,000 rpm, washed twice with deionized water, then lyophilized.

### 4.5. Quercetin-Loaded Chitosan Nanoparticles Characterization

The unhydrated diameter and morphology of QUE.NPs were detected using scanning electron microscopy (SEM) (XL-30 ESEMFEG SEM, FEI Company, USA). SEM images (n = 3) were analyzed using image analysis software (ImageJ, National Institutes of Health, version 1.5a, ImageJ.nih.gov) to determine the average particle diameters of 500 particles.

Zeta potential, polydispersity index (PDI), and dynamic light scattering were evaluated to determine nanoparticle surface charge, size distribution, and hydrodynamic diameter. One mL of NP solution in deionized water (diH_2_O) was analyzed in triplicate using Zetasizer Nano ZS90 (Malvern, UK).

Encapsulation efficiency of CS.Qu.NPs was determined by measuring the amount of unloaded quercetin in the supernatant after centrifugation in the fabrication process [99,100]. Quercetin quantity was determined using a microplate spectrophotometer at 370 nm and calculated from a standard curve of known quercetin concentrations.

$$EE\%=\frac{Total\;amount\;of\;quercetin-Total\;amount\;of\;free\;quercetin\;in\;supernatant}{Total\;amount\;of\;quercetin}\times 100$$

In vitro, the release of quercetin was evaluated by gentle agitation of NPs in phosphate-buffered saline (pH 7.4) at 37 °C, as earlier reported [99,101]. Samples were gathered at fixed time points (1, 2, 4, 8, 24 hrs), and the quantity of quercetin released from the NPs was calculated as illustrated above.

### 4.6. Experimental Designs

Forty-two adult Wistar male rats (weight, 200–220 g) were housed in clean plastic cages for 7 days before the beginning of the experiment at stable room temperature (24–25 °C) and relative humidity (50–55%) on a light/dark cycle (12:12 hrs) with an unobstructed approach to nutritional elements and water.

The rats were haphazardly separated into six groups (n = 7). Group 1 served as the control and received distilled water orally 3 times per week and was injected with normal saline intraperitoneally (i.p) twice weekly for 4 weeks. Group 2, QUE, received 10 mg/kg of quercetin orally 3 times/week for 4 weeks. Group 3, QUE.NPs, received 10 mg/kg of quercetin nanoparticles 3 times/week for 4 weeks. Group 4, CIS, was injected intraperitoneally with 2 mg/kg of cisplatin twice weekly for 4 weeks. Group 5, CIS + QUE, was treated orally with QUE (10 mg/kg) 3 times/week for 4 weeks and injected intraperitoneally with 2 mg/kg of CIS twice weekly for 4 weeks. Group 6, CIS + QUE.NPs, was treated orally with QUE.NPs (10 mg/kg) 3 times/week for 4 weeks and injected intraperitoneally with 2 mg/kg of cisplatin twice weekly for 4 weeks. The doses of CIS and QUE were selected following the aforementioned studies [102,103]. After the last dose, all groups were submitted for behavioral analysis at 10 a.m. Subsequently, rats were euthanized by cervical dislocation, and then kidneys and testes were collected from all groups and preserved for biochemical, histopathological, and immunohistochemical analysis. All procedures were monitored according to the Veterinary Institutional Animal Care and Use Committee (Vet-IACUC, Approval Number: CU23052022453), Faculty of Veterinary Medicine, Cairo University.

### 4.7. Behavioral Analysis

After administration of the corresponding drugs, rats were subjected to behavioral assessment to detect their emotional state using two behavioral tests, including an open field test and a dark–light activity box. These tests were carried out as described in previous studies [104,105]. In the open field test, each rat was monitored for 3 min for their locomotor and exploratory activities, represented by the number of crossings and rearing frequencies in an open arena (70 × 70 × 35 cm) divided into 16 squares. Next, rats were transferred to the dark–light activity box (45 × 45 cm) to detect their anxious state; the box is divided into two chambers (dark and light) separated by a barrier with a sliding door. The rat was positioned in the bright chamber and allowed to explore for 5 min. The percentage of the light chamber and dark chamber durations were recorded. All the behavioral devices were cleansed and stirred with 70% alcohol between each rat to remove fecal matter and urine spots and to remove the olfactory cues for the subsequent rat.

### 4.8. Biochemical Analysis

#### 4.8.1. Assessment of Oxidative Stress in Both Testicular and Renal Tissue Homogenates Using HPLC

Testicular and kidney tissues were weighted and homogenized in 0.05 M PBS (pH 7) using a polytron homogenizer, then centrifuged at 10,000 rpm for a span of 20 min at 4 °C. The supernatant was collected and transferred to −80 °C until biochemical analysis.

Thiol compounds of oxidized and reduced glutathione (GSSG and GSH) in testicular and kidney homogenates were assessed using HPLC (Agilent HP 1200 series, Fort Collins, CO, USA, Cat: WAT027324). This system was set with a quaternary pump, a column oven, a Rheodine injector with a 20 μL loop, and a UV variable wavelength detector. The analytical column employed was a μBondapak C18 Column with a particle size of 10 μm and a pore size of 125 Å. Its dimensions were 3.9 mm × 300 mm. The reference standards for glutathione (oxidized and reduced) were obtained from (Sigma Chemical, Denver, CO, USA) and then data were expressed as μmol/g tissue [63,106].

For lipid peroxidation study, testicular and renal malondialdehyde (MDA) levels were analyzed using an HPLC apparatus. Procedures were performed according to [107]. Briefly, the analytical column employed was a Supelcosil C18 column with a particle size of 5 μm and a pore size of 120 Å. Its dimensions were 250 × 4.6 mm (Catalog Number: 58298). Supernatants were used for determination of MDA, and the MDA standard was prepared by dissolving 25 μL of 1,1,3,3-tetraethoxypropane (TEP) in 100 mL of water to obtain a 1 mM stock solution. To achieve a concentration of 20 nmol/mL TEP, 1 mL of the TEP stock solution was hydrolyzed in 50 mL of 1% sulfuric acid and incubated for 2 h at room temperature, which was further diluted by adding 1% sulfuric acid, resulting in a final concentration of 1.25 nmol/mL

Superoxide dismutase (SOD) was measured for the study of the enzymatic antioxidant status of homogenized testicular and renal tissue. Superoxide dismutase (SOD) activity was analyzed in 2 min intervals. The rate of pyrogallol autoxidation was taken from the decrease in A420 nm per min at 37 °C. Activity was expressed as the amount of enzyme that inhibits the auto-oxidation of pyrogallol and was expressed as % inhibition of pyrogallol oxidation (U = amount of enzyme which inhibits 50% pyrogallol auto-oxidation). Total SOD activity was expressed as U/mg protein. The Bradford method was utilized to measure the total protein content of the supernatants.

#### 4.8.2. Determination of Serum Total Testosterone Testicular IL-10 Content Using an Enzyme-Linked Immunosorbent Assay (ELISA)

Total serum testosterone level and testicular interleukin-10 (IL-10, catalog number SEA056Ra, Cloud-Clone, Houston, TX, USA) were assessed using an ELISA kit, and analysis was performed in accordance with the manufacturer’s instructions.

#### 4.8.3. Sperm Concentration, Viability, and Morphology

Epididymal sperm concentration, viability, and abnormalities of sperm were determined as previously described [108]. Morphological abnormalities of a total of 100 sperm were evaluated per sample. Spermatozoa with malformed heads, detached heads, coiled tails, and curved tails were considered abnormal [109].

#### 4.8.4. Assessment of Serum Kidney Functions

Urea, creatinine, sodium, and potassium levels were detected in serum using specific diagnostic kits (Biodiagnostic, Giza, Egypt, CAT. No. SO 19 10 and CAT. No. PT 18 20).

#### 4.8.5. Assessment of Renal Interleukin-18 and Kidney Injury Molecule-1 Using ELISA

Rat IL-18 (Interleukin 18, Cat. No. E-EL-R0567, Elabscience, Houston, TX, USA) and rat KIM-1 (HAVCR1/KIM-1, catalog number LS-F27504, LSBio, Seattle, WA, USA) were assessed in renal homogenates using ELISA kits.

### 4.9. Histopathological Analysis of Renal and Testicular Tissue

The right kidneys and testis from all groups were fixed in formalin, inserted in paraffin blocks, sliced at a thickness of 4–5 μm, and stained with H&E according to [110]. Slides were inspected by using a BX43 Olympus, Central Valley, Pennsylvania, USA, light microscope. Kidney damage scores were assessed as stated by [111]. Briefly, the evaluation of kidney damage severity was based on the existence of the following criteria: interstitial infiltration of inflammatory cells, degeneration of tubular epithelium, degeneration of glomeruli, necrosis of tubular epithelium, necrosis of glomeruli, and interstitial fibrosis. Each item was scored as follows: 0 = normal, 1 ≤ 25%, 2 = 25–50%, 3 = 50–75%, and 4 ≤ 75%. The total score was obtained by summing the entire score of each lesion (6 fields for each rat/200×).

Histological damage of testicular tissues was assessed by applying Cosentino’s grade [112], as follows: 1 = normal structure; 2 = less organized, fragmented germinal cells, and compactly arranged seminiferous tubules; 3 = pyknotic sloughed germinal cells and unclear seminiferous tubule borders; and 4 = necrotic germinal cells with damaged seminiferous tubules.

Moreover, spermatogenesis was evaluated according to Johnsen’s scoring system, as follows: 1 = absence of both germ and Sertoli cells; 2 = absence of germ cells but presence of Sertoli cells; 3 = presence of spermatogonia only; 4 = presence of little spermatocytes only; 5 = presence of abundant spermatocytes without the existence of spermatozoa or spermatids; 6 = presence of few spermatids only; 7 = presence of several spermatids but absence of spermatozoa; 8 = presence of a few spermatozoa; 9 = copious spermatozoa present, but the germinal epithelium is disorganized; 10 = fulfilled spermatogenesis and intact tubules (20 tubules/rat/200×) [113].

### 4.10. Immunohistochemical Staining of Bcl-2, Bax, and PCNA of Renal and Testicular Tissue

Deparaffinized kidney and testis sections were rehydrated and rinsed three times with phosphate-buffered saline (PBS) containing 3% H_2_O_2_ to block endogenous peroxidase activity. Afterward, slides were incubated overnight with a monoclonal anti-Bcl-2, anti-Bax, or anti-PCNA (1:100, Sigma Chemical Co., St. Louis, MO, USA) to expose anti-apoptotic, pro-apoptotic, and proliferation activity, respectively. Subsequently, sections were washed three times in PBS and kept with secondary antibody for 30 min. A total of 2 mL of DAB-chromogen-substrate was added on slides for 15 min to augment the color reaction; counterstaining with hematoxylin stain was performed. Positive results were expressed as area percentage utilizing ImageJ software. For calculation of the Bax/Bcl-2 ratio, Bax levels were divided by Bcl-2 for each rat.

### 4.11. Statistical Analysis

Data were exhibited as mean ± standard error (SEM) and analyzed by applying one-way analysis of variance (ANOVA) followed by Tukey post hoc test at *p* < 0.05 using GraphPad Prism software (Version 6, San Diego, CA, USA). Histopathological scores were expressed as median ± SEM using the Kruskal–Wallis test, followed by Dunn’s Multiple Comparison.

## 5. Conclusions

Presumably, CIS promotes emotional disturbance and tissue injury via oxidative stress status, which can lead to several redox-sensitive signaling pathways, followed by an upgrade in pro-inflammatory mediators that exaggerate the cytotoxic effect and, eventually, apoptosis. Yet, QUE and its nano formulation curbed cisplatin-induced renal and testicular injury through antioxidant, anti-inflammatory, and antiapoptotic activities. Herein, it can be concluded from the present study that QUE nanoparticles could enhance QUE accessibility and exert more potent protection than QUE in the setting of CIS administration.

## 6. Limitations of Our Study

While this study provides valuable insights into enhancing the protective effects of quercetin through a chitosan nanoparticle delivery platform, there are certain limitations that need to be acknowledged. These limitations include investigation of the distribution and concentration of quercetin-loaded nanoparticles compared to free quercetin within kidney and testicular tissues, demonstration of how liposomal encapsulation enhances the bioavailability of quercetin, and evaluation of long-term safety and potential side effects of quercetin-loaded nanoparticles. Addressing these issues in future research will likely improve the applicability and impact of the study’s outcomes.

## Figures and Tables

**Table 1:** Quercetin and nano-quercetin restore kidney functions of CIS-treated rats. Data are expressed as mean ± SEM (n = 7). * Significant difference from the control group, ** significant difference from the CIS group, *** significantly different from the CIS + QUE group.

Groups	Parameters			
	Creatinine (mg/dL)	Urea (mg/dL)	Potassium-K^+^ (mEq/L)	Sodium-Na+ (mEq/L)
Control	0.81 ± 0.052	44.29 ± 1.11	3.45 ± 0.041	149.0 ± 3.85
QUE	0.81 ± 0.035	42.85 ± 0.96	3.26 ± 0.029	154.4 ± 4.63
QUE.NPs	0.83 ± 0.054	42.87 ± 1.26	3.43 ± 0.064	151.1 ± 4.99
CIS	1.31 ± 0.042 * (*p* < 0.0001)	57.52 ± 1.27 * (*p* < 0.001)	2.50 ± 0.039 * (*p* < 0.001)	210.0 ± 5.90 * (*p* < 0.001)
CIS + QUE	1.10 ± 0.027 ** (*p* < 0.05)	54.39 ± 1.04 ** (*p* < 0.05)	2.42 ± 0.034 **	201.1 ± 5.92
CIS + QUE.NPs	0.94 ± 0.059 *** (*p* < 0.05)	49.30 ± 0.87 *** (*p* < 0.01)	2.90 ± 0.051 *** (*p* < 0.001)	180.0 ± 3.77 *** (*p* < 0.01)

## Data Availability

The authors declare that the data supporting the findings of this study are available within the paper. Should raw data files be needed in another format, they are available from the corresponding author upon reasonable request. Source data are provided in this paper.

## Abbreviations

Bax: pro-apoptotic marker; Bcl-2: anti-apoptotic marker; CIS: cisplatin; GSH: reduced glutathione; GSSG: oxidized glutathione; IL: interleukin; MDA: malondialdehyde; PCNA: proliferating cell nuclear antigen; QUE.NPs: quercetin-loaded chitosan nanoparticles; QUE: quercetin; SOD: superoxide dismutase.
