# Correction: Bakr et al. Efficacy of Quercetin and Quercetin Loaded Chitosan Nanoparticles Against Cisplatin-Induced Renal and Testicular Toxicity via Attenuation of Oxidative Stress, Inflammation, and Apoptosis. *Pharmaceuticals* 2024, *17*, 1384

**DOI:** 10.3390/ph18030348

**Published:** 2025-02-28

**Authors:** Alaa F. Bakr, Riham A. El-Shiekh, Mohamed Y. Mahmoud, Heba M. A. Khalil, Mohammad H. Alyami, Hamad S. Alyami, Omneya Galal, Dina F. Mansour

**Affiliations:** 1 Department of Pathology, Faculty of Veterinary Medicine, Cairo University, Giza 12211, Egypt; 2 Department of Pharmacognosy, Faculty of Pharmacy, Cairo University, Cairo 11562, Egypt; riham.adel@pharma.cu.edu.eg; 3 Department of Toxicology and Forensic Medicine, Faculty of Veterinary Medicine, Cairo University, Giza 12211, Egypt; mohamed_yehia@cu.edu.eg; 4 Department of Veterinary Hygiene and Management, Faculty of Veterinary Medicine, Cairo University, Giza 12211, Egypt; heba.ali315@gmail.com; 5 Faculty of Veterinary Medicine, King Salman International University, South Sinai, Ras Sudr 43312, Egypt; 6 Department of Pharmaceutics, College of Pharmacy, Najran University, Najran 66462, Saudi Arabia; mhalmansour@nu.edu.sa; 7 Department of Pharmacology and Toxicology, Faculty of Pharmacy, Ahram Canadian University, Giza 12581, Egypt; ominagalal@gmail.com; 8 Department of Pharmacology, Medical Research and Clinical Studies Institute, National Research Centre, Cairo 12622, Egypt; dinafmansour@gmail.com; 9 Department of Pharmacy, Faculty of Pharmacy, Galala University, Attaka, Suez 43511, Egypt

**Error in Figure**

In the original publication [1], there was a mistake in Figure 8 as published. This mistake concerned the mislabeling of histopathological images in the control groups of Figure 8 (Bcl2-a&b). The corrected Figure 8 appears below. The authors state that the scientific conclusions are unaffected. This correction was approved by the Academic Editor. The original publication has also been updated.

## Figures and Tables

**Figure 8:**
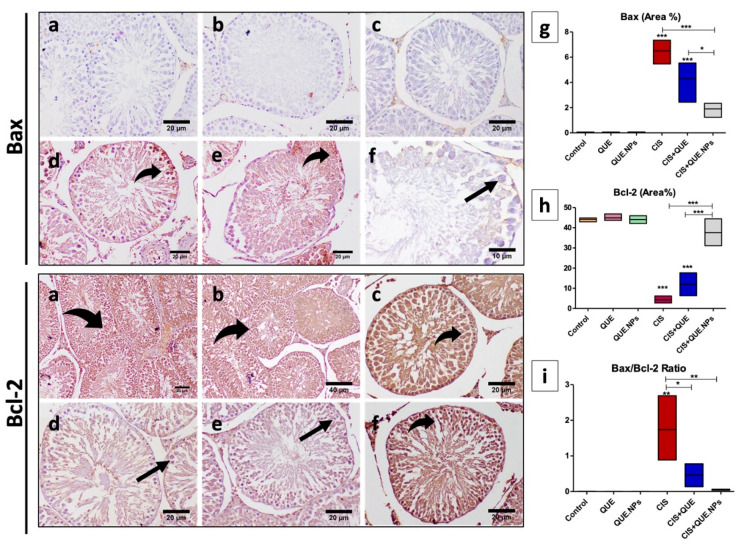
Immunohistochemical staining of testicular tissue with Bax and Bcl-2. (**a**) control group; (**b**) QUE group; (**c**) QUE.NPs group; (**d**) CIS group; (**e**) CIS + QUE group; (**f**) CIS + QUE.NPs group; (**g**) Bax (area%); (**h**) Bcl-2 (area%); (**i**) Bax/Bcl-2 ratio. Data are expressed as mean ± SEM (n = 7). Statistical difference: * *p* < 0.05, ** *p* < 0.01, and *** *p* < 0.001. Remarkable marks shown in the figure are as follows: strong expression (curved arrow) and weak expression (black arrow).
